# Successful surgical excision of primary right atrial angiosarcoma

**DOI:** 10.1186/1749-8090-6-47

**Published:** 2011-04-09

**Authors:** Wobbe Bouma, Chris PH Lexis, Tineke P Willems, Albert JH Suurmeijer, Iwan CC van der Horst, Tjark Ebels, Massimo A Mariani

**Affiliations:** 1 Department of Cardiothoracic Surgery, University Medical Center Groningen, the Netherlands; 2 Department of Cardiology, University Medical Center Groningen, the Netherlands; 3 Department of Radiology, University Medical Center Groningen, the Netherlands; 4 Department of Pathology, University Medical Center Groningen, the Netherlands

## Abstract

Primary cardiac angiosarcoma is a rare and aggressive tumor with a high incidence of metastatic spread (up to 89%) at the time of diagnosis, which restricts the indication for surgical resection to a small number of patients. We report the case of a 50-year old Caucasian woman with non-metastatic primary right atrial angiosarcoma, who underwent successful surgical excision of the tumor (with curative intent) and reconstruction of the right atrium with a porcine pericardial patch. However, after a symptom-free survival of five months the patient presented with bone and liver metastases without evidence of local tumor recurrence.

## Introduction

Angiosarcomas, although rare, are the most common primary malignant neoplasms of the heart [1,2]. Although symptoms are often nonspecific and absent for a long time, primary cardiac angiosarcomas (PCAs) may present abruptly at which point there is often already metastatic spread [2,3]. This restricts the indication for surgical resection to a small number of patients.

In this report we describe the case of a 50-year old Caucasian woman with non-metastatic primary right atrial angiosarcoma, who underwent successful surgical excision of the tumor and reconstruction of the right atrium with a porcine pericardial patch. Surgical resection was performed with curative intent and resection margins were free of tumor cells. However, after a symptom-free survival of five months the patient presented with bone and liver metastases without evidence of local tumor recurrence.

### Case report

A 50-year old female presented with shortness of breath, chest and shoulder pain, and pericardial effusion. Pericardiocentesis yielded 950 ml of pericardial fluid, which was sent for biochemical, microbiological, and cytological analysis. The diagnosis was inconclusive and she was treated for presumed idiopathic recurrent pericarditis with prednisone. Five months later she presented with clinical signs of subacute cardiac tamponade. After pericardiocentesis she quickly recovered, however, pericardial fluid analysis again remained inconclusive. Three months after this second episode transthoracic echocardiography (TTE) revealed a tumor in the right atrium. The patient was then referred to our institution for further evaluation.

**Figure 1:**
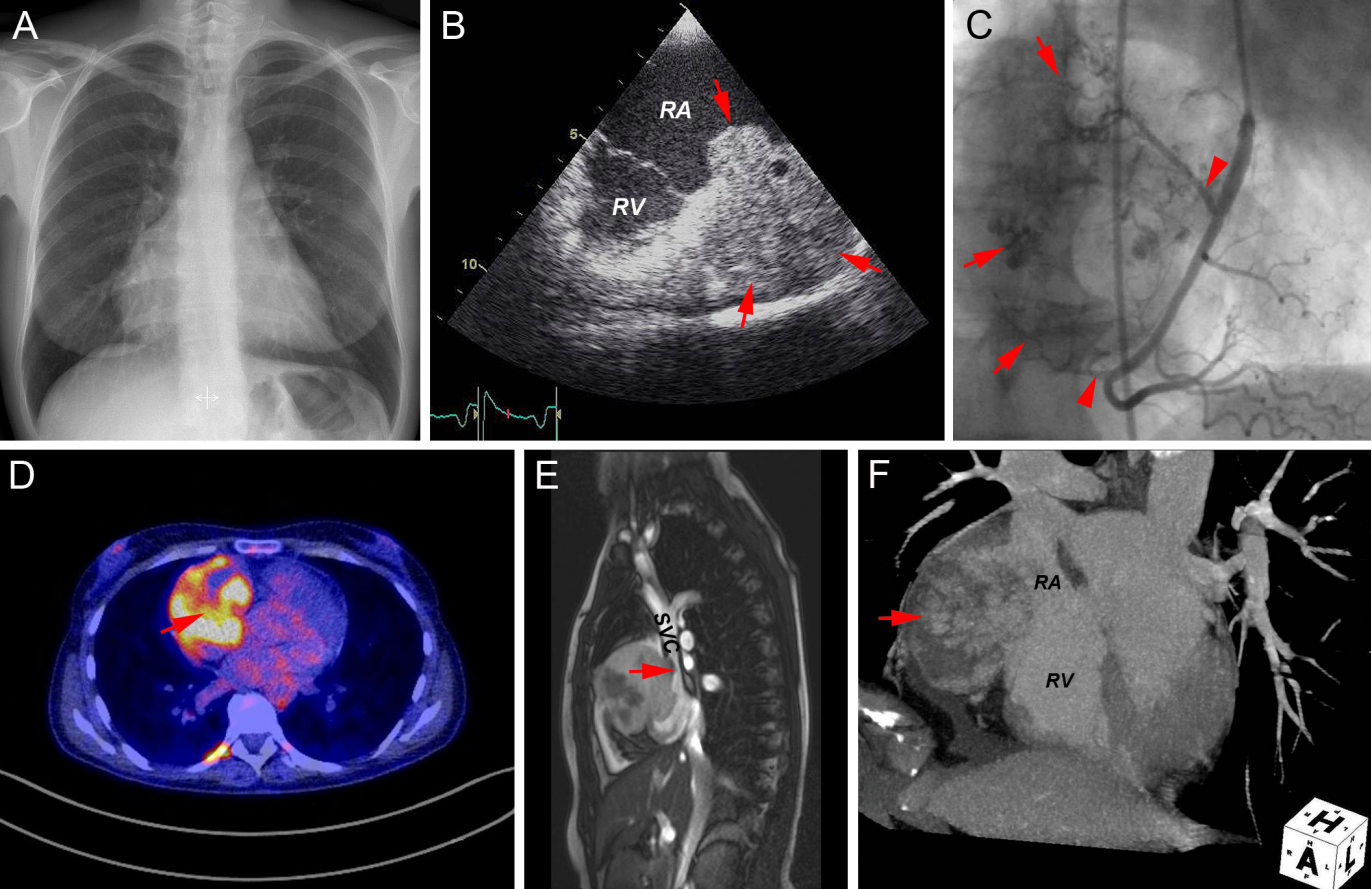
**Preoperative imaging of primary right atrial angiosarcoma**. A. The chest X-ray shows enlargement of the right atrial border (the cardiothoracic ratio is 0.56). B. Transthoracic echocardiography (TTE) (two chamber view of the right heart); confirms pericardial effusion, and shows a giant mass (51 × 44 mm) that infiltrates the right atrial free wall and that protrudes into the right atrium (red arrows). C. Contrast angiogram of the right coronary artery (right anterior oblique projection) showing the right coronary artery and two right atrial branches (red arrowheads) with several small areas of abnormal contrast enhancement, characteristic of a "tumor blush" (red arrows) (representing new vessel formation feeding the angiosarcoma). D. FDG (fluorodeoxy-glucose-18) PET-CT scanning (transverse section, four chamber view) to assess metabolic activity reveals hypermetabolic uptake of FDG in the right atrium (red arrow), consistent with malignancy. Paravertebrally, there is physiologic brown fat activity. E. Turboflash 2D cine MRI (sagittal section, through the superior vena cava); large inhomogenous tumor in the lateral wall of the right atrium (diameter of approximately 74 mm), extending into the wall of the superior vena cava (red arrow). F. CT (reconstruction along the cardiac axis); large inhomogenous tumor in the lateral wall of the right atrium, protruding into the right atrium (64 × 53 mm), without invasion of the right ventricular wall (red arrows). *A, coronal plane; L, sagittal plane; H, horizontal plane; RA, right atrium; RV, right ventricle; SVC, superior vena cava*.

A chest X-ray (Figure 1A) showed enlargement of the right atrial border and an electrocardiogram showed normal sinus rhythm with a heart rate of 87 beats per minute.

Transthoracic (TTE) and transesophageal echocardiography (TEE) confirmed pericardial effusion, and showed a giant mass (51 × 44 mm) that infiltrated the right atrial free wall and that protruded into the right atrium (Figure 1B). Left ventricular function was normal and there were no valvular abnormalities.

Coronary angiography of the right coronary artery (Figure 1C) showed two right atrial branches with several small areas of abnormal contrast enhancement, representing new vessel formation feeding the tumor ("tumor blush"). Right heart catheterization revealed normal right heart and pulmonary artery pressures and showed no signs of obstruction of blood flow in the superior and inferior vena cava, in the right atrium and ventricle, or in the pulmonary artery.

Fluorodeoxy-glucose-18 - positron emission tomography - computer tomography (FDG-PET-CT) scanning (Figure 1D) was performed to assess metabolic activity and revealed hypermetabolic uptake of FDG in the right atrium, consistent with malignancy. No metastatic spread to any other organs was seen.

Cardiovascular magnetic resonance imaging (MRI) (Figure 1E) and CT (Figure 1F) showed a large excentric and inhomogenous tumor (74 × 64 × 53 mm) in the right atrial free wall, protruding into the right atrium, compressing the right atrial appendage, and extending into the wall of the superior vena cava. The tumor was in close proximity to the ascending aorta and extended into the right atrioventricular groove, but did not involve the right coronary artery, the right ventricle, or the annulus of the tricuspid valve. Pericardial effusion was identified around the tumor, right atrium, and right ventricle.

CT, MRI, and PET did not reveal any positive lymph nodes or mediastinal or lung involvement. MRI of the brain did not show any cerebral metastases and a normal mammogram excluded primary breast cancer.

**Figure 2:**
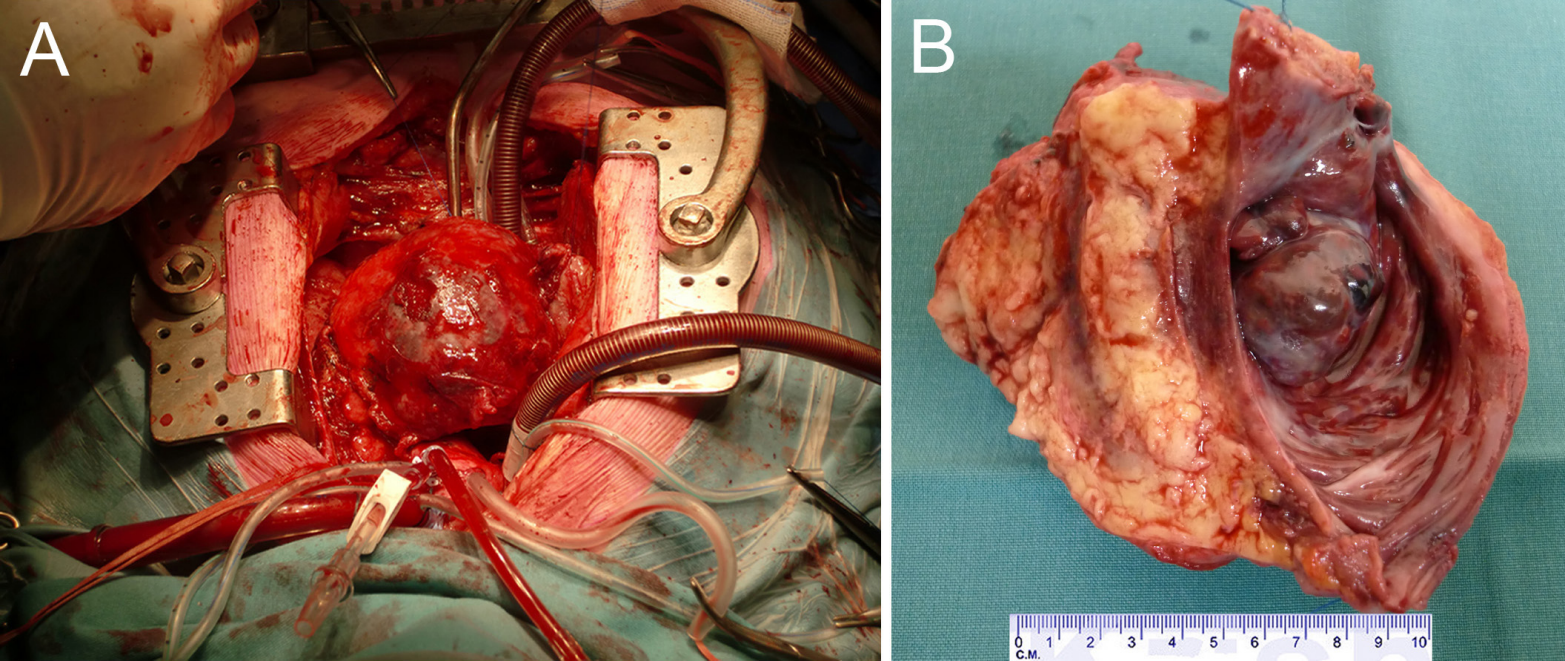
**Macroscopic pathology photographs of primary right atrial angiosarcoma**. A. Intraoperative photograph; initial view of the right atrial tumor during surgery. B. Macroscopic photograph; broadly resected large tumor of the right atrial free wall as seen from inside the right atrium. The tumor protrudes into the right atrium (tumor size: 100 × 70 × 45 mm).

Twenty-two days after the initial diagnosis the patient underwent surgical excision of the tumor (Figure 2A). Histologic examination of a frozen section during surgery revealed that the tumor was malignant (most likely a type of sarcoma). The tumor was resected successfully with curative intent. First, the tumor was dissected from the base of the ascending aorta. Second, the right atrium was opened and the tumor was dissected with a broad margin from the superior vena cava and the septum down to the annulus of the tricuspid valve. A free margin remained above the annulus for reconstruction. Third, the atrioventricular groove was dissected and the right coronary artery, which was adherent to, but not invaded by the tumor, was successfully dissected. All branches feeding the tumor were clipped. The tumor was successfully resected with a tumor-free margin on each side. Finally, the right atrium was reconstructed with a porcine pericardial patch.

**Figure 3:**
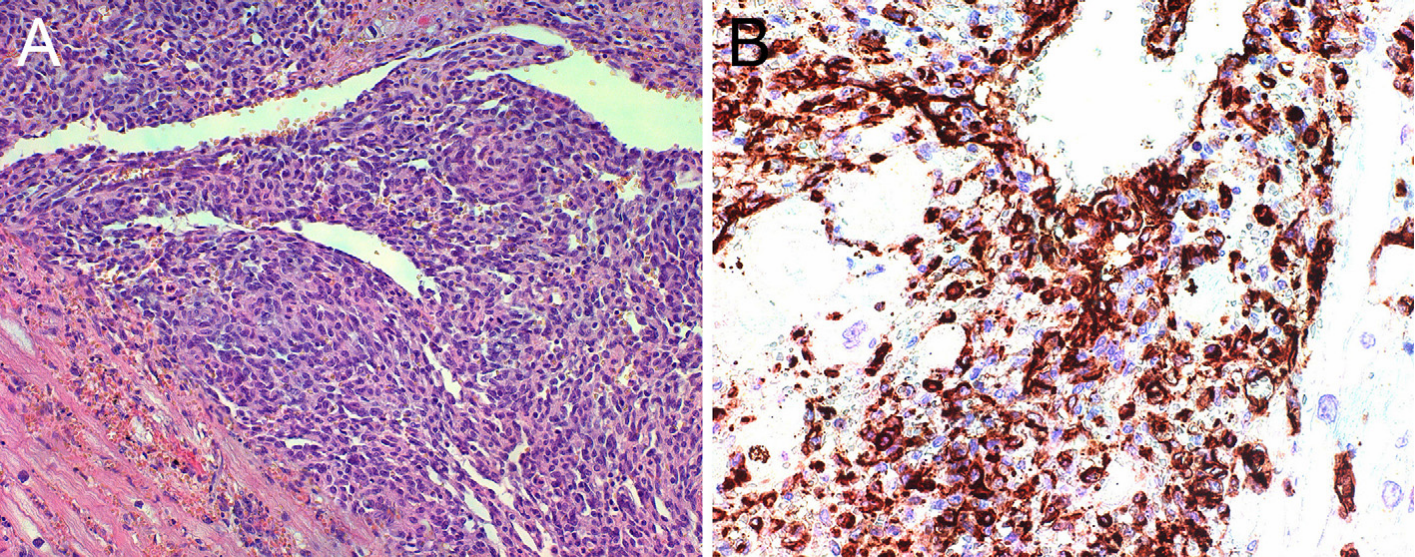
**Microscopic pathology photographs of primary right atrial angiosarcoma**. A. Histologic photomicrograph (HE stain, original magnification 20×); the tumor consists of spindle-shaped cells with pleomorphic nuclei lining anastomosing vascular spaces. Mitotic figures and areas of hemorrhage and necrosis can also be found. These findings support the diagnosis of angiosarcoma. The lower left corner shows myocardial invasion with tumor cells. B. Immunohistochemistry photomicrograph (CD31 stain, original magnification 40×); the tumor cells are positive for the endothelial marker CD31, which confirms the vascular nature of the tumor.

The resected tumor was 100 × 70 × 45 mm in size (Figure 2B). Histopathologic examination (Figure 3A) showed a hemorrhagic and necrotic malignant tumor that invaded atrial myocardium and epicardium. The tumor contained solid areas and anastomosing vascular spaces lined by spindle-shaped cells with pleomorphic hyperchromatic nuclei and brisk mitotic activity. The resection margins were free of tumor cells, but the tumor extended to the epicardial surface with a small margin of less than 1 mm. Immunohistochemically, the tumor cells were positive for the endothelial markers factor VIII-related protein, CD31 and CD34 (Figure 3B), whereas reactivity to podoplanin, smooth muscle actin, desmin, S100 protein, keratins, and EMA was negative. These findings confirmed the diagnosis of primary right atrial angiosarcoma.

Postoperative recovery was uneventful and the patient was discharged on the eleventh postoperative day. A follow-up CT-scan after three months revealed no tumor recurrence. After a symptom-free survival of five months the patient unfortunately presented with bone and liver metastases without evidence of local tumor recurrence. Both chemotherapy and irradiation were started.

## Discussion

Primary cardiac tumors are rare, with an incidence at autopsy from 0.0017% to 0.033% [4]. Metastases are by far the most common cardiac neoplasms; they are 40 times more prevalent than primary cardiac tumors [1]. In adults, approximately 75% of primary cardiac tumors are benign, with myxoma accounting for up to half of cases. The remaining 25% of primary cardiac tumors are malignant, and one-third of those are angiosarcoma [5]. Other primary malignant cardiac tumors include rhabdomyosarcoma, osteosarcoma, leiomyosarcoma, undifferentiated sarcoma, and primary cardiac lymphoma [1]. Primary cardiac angiosarcomas (PCAs) have a tendency to occur in the third to fifth decade and are more common in males [2,3,6]. Ninety percent of the angiosarcomas are located in the right atrium [6]. The lateral (free) wall of the right atrium is the most common site, the septum being spared in most cases [2,3,6].

PCAs may present abruptly with a fulminant clinical course. The clinical signs and symptoms are often nonspecific. Symptoms are often absent for a long time and are related to the cardiac location of the tumor, its size, the degree of myocardial involvement, and the presence of metastases [4,7,8]. Because of the propensity of the tumor to involve the right atrium and pericardium, patients may present with right-sided heart failure, superior vena cava obstruction, and recurrent pericardial effusions or cardiac tamponade [2,8,9]. Dyspnea is the most common presenting symptom; additional symptoms include atypical chest pain, hemoptysis, orthopnea, and nonspecific symptoms such as nausea, emesis, fever, and anorexia [7].

PCAs are aggressive tumors, with metastasis found in 66 to 89% of patients at the time of diagnosis [2,3]. PCAs most commonly metastasize to the lungs, but also occasionally to lymph nodes, bone, liver, brain, bowel, spleen, adrenal glands, pleura, diaphragm, kidneys, thyroid, and skin [10].

The differential diagnosis of a right atrial mass includes benign entities such as myxoma and thrombus and malignant causes such as metastatic involvement of the heart, primary cardiac angiosarcoma and other sarcomas, pericardial mesothelioma, and primary cardiac lymphoma [11].

Echocardiography has become the primary diagnostic technique because of its high degree of accuracy, non-invasiveness, and cost effectiveness [12]. Besides echocardiography, CT, MRI, and PET can be of benefit in the diagnostic work-up [13,14]. These imaging modalities are particularly helpful in defining the extent to which the cardiac tumor infiltrates surrounding structures and when assessing the patient for metastases to other organs. CT and MRI can both show tumor infiltration of the myocardium and direct extension into the pericardium [9]. On CT angiosarcomas appear as irregular lobulated low-attenuation masses that frequently extend to involve the adjacent pericardium and vessels [11]. Cardiac MRI enables the most comprehensive imaging assessment of cardiac neoplasms. In contrast to TTE, cardiac MRI provides improved soft-tissue contrast, tissue characterization, and assessment of mediastinal and lung involvement by the tumor [11]. The addition of imaging with a gadolinium-based contrast agent allows an assessment of the extent of tumor vascularity and further improves the differentiation from surrounding structures [11]. The presence of large blood filled spaces might account for the "cauliflower appearance" (local nodular areas of increased signal intensity interspersed with areas of intermediate signal intensity) [15].

Cytologic examination on fluid obtained by pericardiocentesis rarely yields a conclusive diagnosis [16]. In case of negative pericardial fluid cytology, tissue specimens can be obtained by thoracotomy, TTE or CT guided biopsy [8], or TEE guided transvenous endomyocardial biopsy [17]. However, biopsy is frequently non-diagnostic and carries a considerable risk of cardiac rupture because the right atrial wall is thin [8].

Histopathology defines angiosarcoma as a malignant tumor whose cells display endothelial differentiation [2]. Microscopically, tumor differentiation is reflected by formation of irregular anastomozing vascular spaces. Poorly differentiated angiosarcomas show solid growth. Tumor cells can still have an endothelial morphology or can be spindle-shaped with malignant appearing, hyperchromatic nuclei [18]. Mitotic figures and areas of hemorrhage and necrosis are always present [18]. The diagnosis of cardiac angiosarcoma can be confirmed by additional immunohistochemical staining for endothelial markers, of which CD31 and factor VIII-related protein are most specific [19].

Appropriate evidence-based treatment guidelines have not been established because of the rarity of the tumor [20]. Surgical resection is indicated when no evidence of metastasis exists and when myocardial resection is reparative [13]. The surgical approach is often difficult since PCAs usually are so large at the time of diagnosis that complete resection cannot be achieved. However, even incomplete resection may provide substantial symptom-free survival [21]. In case of extensive infiltration of the right heart requiring partial cardiectomy for complete surgical resection, functional reconstruction may be achieved with a cavopulmonary shunt or Fontan-type operation, excluding the right heart from the circulation [22]. Anatomic and functional reconstruction of the right heart may also be accomplished with a right atrial patch [13], as we have also shown in this report.

Chemotherapy and irradiation were reported not to improve survival [7] and their use is usually limited due to the poor physical condition of the patient. However, a multidisciplinary approach involving surgery, irradiation, adjuvant chemotherapy, and immunotherapy, using interleukin-2, may offer hope for increased survival in selected patients [23]. Cardiac transplantation has been performed in a few patients, however, with a poor outcome [24]. There is no evidence that cardiac transplantation improves the overall poor prognosis of these patients. Moreover, there is concern about enhancing tumor growth in the setting of immunosuppressive drugs [25].

The prognosis of cardiac angiosarcoma is universally poor: survival ranges from six to nine months, regardless of the treatment chosen [7]. Death results from infiltration of the myocardium, cardiac tamponade, obstruction of flow, and/or distant metastases.

## Conclusions

The high frequency of metastatic spread at the time of diagnosis (up to 89%) combined with the aggressive behaviour of PCAs usually results in disappointing treatment outcomes. However, early detection and aggressive treatment may lead to a more favorable outcome and may extend survival beyond one year. Therefore, when a patient presents with (recurrent) pericardial effusions or when a right-sided cardiac mass is detected, there should always be a high level of suspicion for PCA. Newer imaging modalities, including CT and MRI, can help define the exact location and extent of the tumor and aid in the planning of surgical resections. Due to the rarity of PCA treatment options are at this point limited and not evidence-based.

This case of primary right atrial angiosarcoma in a 50-year old Caucasian woman highlights its nonspecific clinical presentation, the diagnostic delay, the broad spectrum of diagnostic imaging modalities, and the rapid and aggressive natural course of cardiac angiosarcomas. Although successful surgical resection of PCA (with curative intent) in the apparent absence of metastatic spread is possible, there may be micrometastases at the time of diagnosis and surgery. In that case, an apparently curative (local) surgical resection may provide substantial symptom-free survival, but it has little influence on the rapid and aggressive natural course of PCA.

## Informed consent

Written informed consent was obtained from the patient for publication of this case report and any accompanying images. A copy of the written consent is available for review by the Editor-in-Chief of this journal.

## Competing interests

The authors declare that they have no competing interests.

## Authors' contributions

WB and CL collected the data and wrote the manuscript. TW, AS, IH, TE, and MM participated in the design of the manuscript and they revised and critically reviewed the manuscript. All authors read and approved the final manuscript.
